# Urinary stone composition in Germany: results from 45,783 stone analyses

**DOI:** 10.1007/s00345-022-04060-w

**Published:** 2022-06-06

**Authors:** Roswitha Siener, Helena Herwig, Jakob Rüdy, Reinhold M. Schaefer, Philipp Lossin, Albrecht Hesse

**Affiliations:** 1 Department of Urology, University Stone Center, University Hospital Bonn, Venusberg-Campus 1, 53127 Bonn, Germany; 2 Urinary Stone Analysis Center Bonn, Bonn, Germany

**Keywords:** Stone composition, Urolithiasis, Kidney stones, Age, Sex, Epidemiology

## Abstract

**Purpose:**

Stone composition can provide valuable information for the diagnosis, treatment and recurrence prevention of urolithiasis. The aim of this study was to evaluate the distribution of urinary stone components and the impact of different crystal forms according to gender and age of patients in Germany.

**Methods:**

A total of 45,783 urinary stones submitted from 32,512 men and 13,271 women between January 2007 and December 2020 were analyzed by infrared spectroscopy. Only the first calculus obtained per patient was included in the analysis.

**Results:**

The most common main stone component was calcium oxalate (CaOx) (71.4%), followed by carbonate apatite (CA) (10.2%) and uric acid (UA) (8.3%). Struvite (2.1%), brushite (1.3%), protein (0.5%) and cystine (0.4%) stones were only rarely diagnosed. CaOx (75%) and UA stones (81%) were more frequently obtained from men than women (*p* < 0.001). Weddellite (COD) and uric acid dihydrate (UAD) were more common in younger ages than whewellite (COM) and anhydrous uric acid (UAA), respectively, in both men and women. The ratios of COM-to-COD and UAA-to-UAD calculi were approximately 4:1 and 8:1, respectively. The peak of stone occurrence was between the ages of 40 and 59 years.

**Conclusion:**

Stone composition is strongly associated with gender and age. The peak incidence of calculi in both women and men was in the most active phase of their working life. The distinction between different crystal forms could provide clues to the activity and mechanisms of lithogenesis. Further research is needed in understanding the causative factors and the process of stone formation.

**Supplementary Information:**

The online version contains supplementary material available at 10.1007/s00345-022-04060-w.

## Introduction

Urolithiasis is among the most common urologic diseases and imposes a significant burden on the healthcare system [1, 2]. The prevalence of urinary stone disease is estimated to be nearly 5% in Germany and 10% in the United States [3, 4]. Despite the availability of excellent treatment modalities, the recurrence rate of urinary stones is reported to be up to 50% after 10 years [3, 5]. Exact compositional stone analysis is the most important laboratory diagnostic procedure and a crucial prerequisite for an effective treatment and recurrence prevention of urolithiasis [6–8].

Previous studies on the distribution of urinary stone types in different countries revealed that calcium oxalate (CaOx) was the most frequent stone constituent followed by carbonate apatite (CA) and uric acid (UA) [9–11]. CaOx occurs in two different hydrate forms, whewellite (calcium oxalate monohydrate; COM) and weddellite (calcium oxalate dihydrate; COD). Due to its hardness, COM calculi are considered to respond poorly to disintegration by ESWL [12]. Likewise, UA can be present in two crystal species, anhydrous uric acid (UAA) and uric acid dihydrate (UAD) [13]. Although distinguishing between different hydrate forms may have implications for stone therapy and could provide valuable information about the etiology of stone formation, data on the occurrence and characteristics of COM, COD, UAA and UAD are scarce. While a single study based on 27,980 calculi reported on the distribution of COM and COD by sex and age [9], data on gender and age-related aspects of UAA and UAD are lacking. Therefore, data from 45,783 stone analyses were collected with the aim of evaluating the distribution of urinary stone components and different crystal forms according to age and gender of patients.

## Materials and methods

### Stone analyses

In total, 45,783 urinary stones submitted for analysis to the Urinary Stone Analysis Center Bonn and the University Stone Center of the Department of Urology, University Hospital Bonn, from 2007 to 2020 were evaluated. Urinary stone samples were obtained from all over Germany. Stones were collected after spontaneous passage, surgery, chemolysis, lithotripsy or instrumental procedures. To avoid overestimation of any stone type by multiple stones from the same patient, only the first calculus obtained per patient was included in the analysis. Patients with incomplete data in terms of age or sex were excluded from the study.

Each stone was analyzed using a standard operating procedure. The stones were dried at 37 °C and then crushed into a fine, homogenized powder using an agate mortar. Analysis was performed by Fourier transform infrared spectroscopy (FTIR) (Perkin Elmer, Waltham, MA, USA). The evaluation of the percentage of stone constituents was performed by comparing the graphs of the stone samples to a computerized library of reference spectra of single and mixed constituents. Each evaluation was examined by qualified and trained personnel and double-checked to ensure an accurate analysis. Laboratory quality certification was available for stone analysis. The FTIR technique is currently considered as the gold standard for routine clinical analysis of stone composition [10].

### Stone classification

Mineral components accounting from 5% (weight-%) were counted. Stones containing a majority of > 50% of a single constituent were classified as such. Calculi without a main component > 50% were classified as being mixed. Stones containing any brushite were placed in the brushite group. Stones containing any cystine were classified as cystine. Materials unlikely having an origin in the human urinary tract, such as cellulose or wax, were classified as artifacts.

### Statistical analyses

Categorical variables are presented as percentages. The effect of age and gender on different stone types was assessed by the Chi-squared test. Fisher’s exact test was used if the Chi-squared test was not applicable. The significance level was considered as *p* < 0.05. Statistical analysis was performed using SPSS for Windows, version 27.

## Results

### Stone composition

Of the 45,783 urinary stones included in the analysis, 71.4% were composed mostly of CaOx, followed by CA (10.2%) and UA (8.3%) (Table 1). Struvite (2.1%), brushite (1.3%), protein (0.5%), cystine (0.4%) and urate (0.3%) stones were only rarely diagnosed. Only four 2,8-dihydroxyadenine stones were submitted. In total, 56 samples contained silicate, 28 samples consisted of calcite, 16 stones contained drug metabolites and 46 samples were classified as artifacts. A total of 2216 stones (4.8%) were mixed stones containing no majority of a component.

**Table 1:** Distribution of stone components (*n* = 45,783)

Main component	Total number	%	Men number	%	Women number	%	*p*	*M*/*F*
Calcium oxalates								
Whewellite	26,017	56.8	19,518	60.0	6499	49.0	< 0.001	3.00
Weddellite	6685	14.6	4921	15.1	1764	13.3	< 0.001	2.79
Phosphates								
Carbonate apatite	4649	10.2	2214	6.8	2435	18.3	< 0.001	0.91
Brushite	585	1.3	429	1.3	156	1.2	0.231	2.75
Struvite	959	2.1	415	1.3	544	4.1	< 0.001	0.76
Other phosphates	163	0.4	65	0.2	98	0.7	< 0.001	0.66
Uric acid and urates								
Uric acid anhydrous	3390	7.4	2755	8.5	635	4.8	< 0.001	4.34
Uric acid dihydrate	414	0.9	344	1.1	70	0.5	< 0.001	4.91
Ammonium urate	83	0.2	49	0.2	34	0.3	0.022	1.44
Sodium/potassium urate	38	0.1	28	0.1	10	0.1	0.858	2.80
Protein								
Protein	245	0.5	158	0.5	87	0.7	0.029	1.82
Genetically determined stones								
Cystine	189	0.4	120	0.4	69	0.5	0.028	1.74
2,8-Dihydroxyadenine	4	0.01	2	0.02	2	0.01	0.330	1.00
Xanthine	0	–	0	–	0	–	–	–
Others								
Artifacts	46	0.1	28	0.1	18	0.1	0.141	1.56
Silicate	56	0.1	29	0.1	27	0.2	0.002	1.07
Calcite	28	0.1	15	0.05	13	0.1	0.058	1.15
Drugs	16	0.03	10	0.05	6	0.03	0.422	1.67
Without main component								
Without main component	2216	4.8	1412	4.3	804	6.1	< 0.001	1.76
Total	45,783	100	32,512	100	13,271	100	< 0.001	2.45

*P* value for comparison between genders

*M/F* male-to-female ratio

### Gender

The majority of stones was obtained from men (71.0%) as opposed to women (29.0%) resulting in a male-to-female ratio of 2.45 (Table 1). CaOx (75.1% versus 62.3%) was the most common main component in both men and women, followed by CA (18.3%), UA (5.3%) and struvite (4.1%) in women and by UA (9.6%) and CA (6.8%) in men. CaOx (75%) and UA stones (81%) were more frequently obtained from men than women (*p* < 0.001). Of CaOx and UA stones, COM (79.9% and 78.7%, respectively) and UAA (88.9% and 90.1%, respectively) were substantially more common than COD (20.1% and 21.3%, respectively) and UAD (11.1% and 9.9%, respectively) stones in men and women.

### Age

**Fig. 1:**
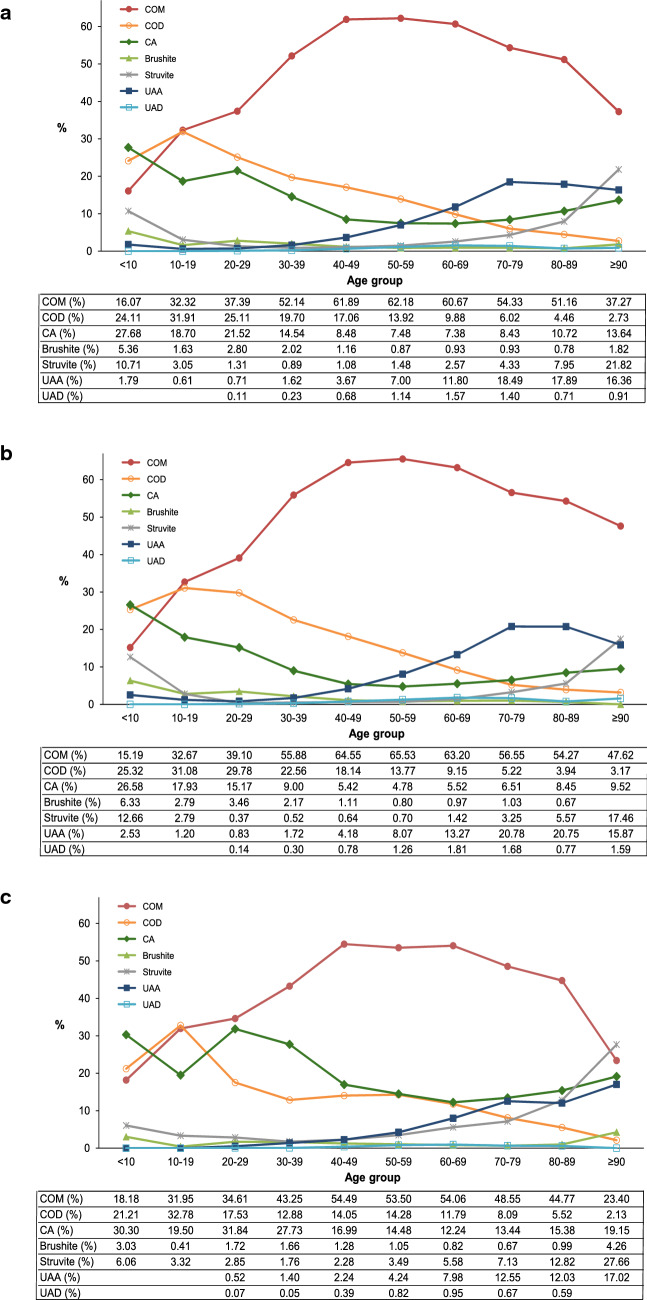
Association of gender and age with stone type. **a** Total **b** Men **c** Women

The peak incidence of stones in both women (42.4%) and men (46.3%) were between the ages of 40 and 59 years, although this age group comprised only 30% of the general population in Germany [14] (supplementary Table 1). Age trends in stone distribution were similar in both genders for most stone types (Fig. 1a–c). CaOx was the most common main stone component in both genders and all age groups. While COD was second most frequently obtained from patients < 10 years old, COM was the relatively predominant main stone constituent between 20 and 89 years of age in both genders. The occurrence of UA stones increased strongly in men and women ≥ 60 years old, whereas CA stones were more frequently observed in patients under 40 years of age. Struvite stones were most common in the youngest and oldest age groups.

### Stone constituents related to age and gender

**Fig. 2:**
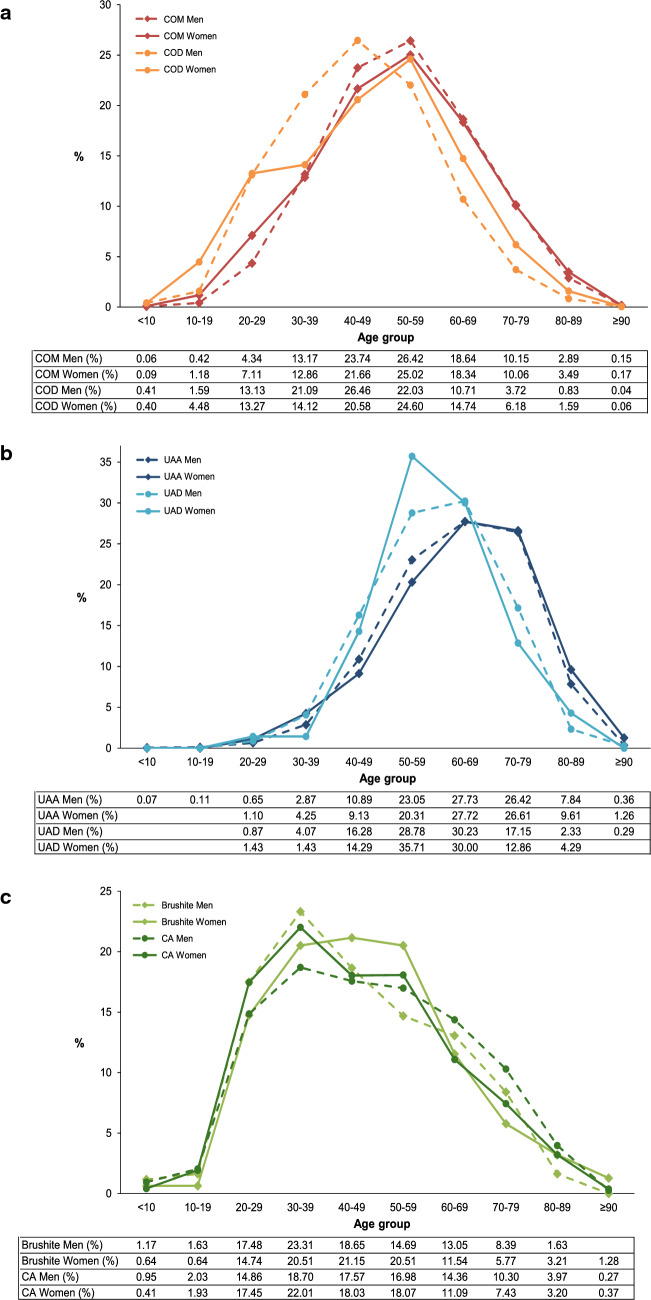
Percentage age distribution of stone types in men and women. **a** Whewellite and weddellite **b** Uric acid anhydrous and uric acid dihydrate **c** Carbonate apatite and brushite

The percentage age distribution of different stone constituents in both genders is shown in Fig. 2a–c. Of the two hydrate forms of CaOx, COD was more frequent in women < 40 years old and in men < 50 years of age (Fig. 2a). In men, the peak incidence of COD stones occurred ten years earlier compared to COM. Of the two crystal forms of UA, an earlier age peak of UAD of ten years was observed in women and a higher occurrence in men < 69 years (Fig. 2b). The age peak of CA stones was between 30 and 39 years in both men and women (Fig. 2c). The maximum occurrence of brushite stones was between 30 and 39 years in men and between 30 and 59 years in women.

## Discussion

Accurate compositional stone analysis using a reliable laboratory method is a crucial basis for effective diagnosis and treatment of urolithiasis [6, 7]. The distribution of stone components, the frequency of different hydrate forms and the impact of demographic factors could provide additional information about the etiology, therapy and recurrence prevention of stone disease. The current study presents the most recent data of urinary stone characteristics in Germany. CaOx was the most common main stone constituent in both genders, with 71.4% of all submitted calculi. The incidence of CaOx stones was higher in men. The current data confirm the high proportion of CaOx stones and the preponderance of men reported in other large series of patients [9–11]. Unfortunately, in a previous large series of stone analyses in Germany, calcium-containing calculi were not differentiated into CaOx and calcium phosphate stones [15].

The current study provided the largest database to date of urinary stones distinguishing between age and gender-related aspects of both hydrate forms of CaOx. The majority of COM and COD was obtained from men, corresponding to the greater incidence of CaOx stones in men. COM occurred substantially more frequent compared to COD stones with a ratio of approximately 4:1 in both genders. Moreover, COD was more common than COM until young adulthood in both genders, resulting in an earlier age peak, whereas the proportion of COM was higher in older age groups. A higher proportion of COM compared to COD stones in both genders and a preponderance of COD in younger age groups has also been reported in a prior study based on 27,980 calculi [9], while other large series of patients did not differentiate between COM and COD [10, 11]. The distinction between COM and COD may point to possible formation conditions of the two hydrate forms of CaOx. The propensity to develop COM or COD has been related to specific urinary risk factors. Several studies suggested that hyperoxaluria could contribute to the formation of COM [16, 17], while hypercalciuria might favor the formation of COD [16–18]. The decline in the frequency of COD calculi with increasing age has been explained with a decrease in calcium excretion with age [9, 19]. However, no correlation of calcium excretion with age was observed in a recent study of 993 CaOx stone-forming patients [20]. Moreover, a previous study of stone patients found urine chemistry of limited value in distinguishing COM from COD stones [21].

Another explanation for the high ratio of COM-to-COD could be the formation process of the two hydrate forms of CaOx. Thermodynamically, COM is the more stable crystal form, whereas COD is metastable and is considered as the primary phase of CaOx stone formation [13, 22]. The conversion of COD to COM in urinary stones has been demonstrated convincingly [13, 22, 23]. Evidence suggests that CaOx stones undergo repeated events of dissolution and recrystallization during growth within the kidney [23, 24]. These findings might also explain a higher detection of COM with progressive age. It is hypothesized that COD stone formation at younger age possibly occurs due to a fast emerging process with a lack of time for dissolution and recrystallization processes. Urinary inhibitors that hinder remodelling processes at younger ages or promotors that initiate transition processes in older age are also conceivable. Osteopontin, an inhibitor of CaOx stone formation, has been reported to modify CaOx crystallisation kinetics towards the formation of COD rather than COM [25]. Decreasing blood levels of osteopontin with age could favour higher COD proportions with increasing age [26].

In the present study, CA was the second most common stone type in the German population. Both men and women were more susceptible to CA stones at younger ages. These findings are consistent with other studies [9–11]. The causes of CA stone formation include distal renal tubular acidosis, primary hyperparathyroidism and vitamin D supplementation [27]. Although urinary tract infection is not a prerequisite for the formation of CA stones, an infection component associated with alkaline urine favors CA stone formation [27]. Characteristic infection stones, i.e. struvite stones, were only rarely observed in this study, but were most frequent in the youngest and oldest age groups. Factors that predispose to urinary tract infections are, among others, vesicoureteral reflux in children and indwelling urinary catheter in the elderly.

UA stones were the second most common stones in men and the third most frequent stone type in women. UA calculi became more common with increasing age. Previous studies reported a similar age trend [9–11, 15]. Changes in renal function associated with aging, particularly diminishing urine pH [28], might increase urine supersaturation with UA. Furthermore, the increasing prevalence of overweight and insulin resistance is associated with more acidic urine and UA stones [29]. To our knowledge, the present study provided the first results of UA stones distinguishing between gender and age-related aspects of the two hydrate forms of uric acid. UAA stones were substantially more common than UAD calculi with a ratio of approximately 8:1. The only large series of patients to date that differentiated between the two crystalline forms of UA also reported a higher proportion of UAA compared to UAD stones [9]. UAD is exceedingly unstable and is frequently the primary phase of the formation of UA calculi [13]. A similar dehydration process to that described for the two hydrate forms of CaOx has been suggested for the transformation of UAD into the more stable UAA [13, 23].

Exact compositional stone analysis can provide essential information about factors affecting stone formation. The current study presented the largest series of stone analyses to date on the distribution of the different hydrate forms according to age and gender of patients. The distinction between the different hydrate forms of CaOx and UA in stone analysis in the present study is an important step to understanding the specific circumstances of their formation conditions. The formation mechanisms of the different crystal species must be verified to establish effective measures for the treatment and recurrence prevention of these types of stone. Further research is needed in understanding the causative and driving factors and the process of stone formation.

The study has a potential limitation. Clinical data of the patients were not available, other than age, gender, and the referral site submitting the stone. Nevertheless, the very large number of stone analyses available for this report provided valuable information on the distribution of urinary stone types and on sex and age-related predispositions to stone formation. Since the current data confirmed the frequency of the most common stone types reported in previous studies, it can be assumed that the results of the present study, especially on the different hydrate forms of calcium oxalate and uric acid, can be generalized to countries other than Germany. This largest series of stone analyses to date differentiating between age and gender-related aspects of different hydrate forms of stone constituents should give clues to the mechanisms and activity of the process of stone formation.

## Conclusion

The most common stones in Germany were CaOx, followed by CA and UA. The peak incidence of stones was between the ages of 40 and 59 years of patients, i.e., in the most active phase of their working life. A clear predominance of COM and UAA over COD and UAD, respectively, was observed in both genders. The distinction between different crystal forms could provide clues to the activity and mechanisms of the lithogenic process. Understanding the mechanisms of stone formation is crucial for appropriate individualized treatment and recurrence prevention of each patient.

## Supplementary Information

Below is the link to the electronic supplementary material.

Supplementary file1 (DOCX 18 KB)
