# Supplementary material for: Urinary stone composition in Germany: results from 45,783 stone analyses
Source: World J Urol. 2022 Jun 6;40(7):1813–20. doi: 10.1007/s00345-022-04060-w (PMC9236976; doi:10.1007/s00345-022-04060-w)
Supplement: Supplementary file 1 — Supplementary file1 (DOCX 18 KB) [file 345_2022_4060_MOESM1_ESM.docx]

| **Age group** | **Total** |  | **Men** |  | **Women** |  | **M/F** | **Census^a^** |
| --- | --- | --- | --- | --- | --- | --- | --- | --- |
|  | Number | % | Number | % | Number | % |  | % |
| <10 | 112 | 0.2 | 79 | 0.2 | 33 | 0.2 | 2.39 | 8.8 |
| 10-19 | 492 | 1.1 | 251 | 0.8 | 241 | 1.8 | 1.04 | 9.7 |
| 20-29 | 3,504 | 7.7 | 2,169 | 6.7 | 1,335 | 10.1 | 1.62 | 12.0 |
| 30-39 | 6,534 | 14.3 | 4,601 | 14.2 | 1,933 | 14.6 | 2.38 | 12.4 |
| 40-49 | 9,761 | 21.3 | 7,177 | 22.1 | 2,584 | 19.5 | 2.78 | 14.8 |
| 50-59 | 10,909 | 23.8 | 7,870 | 24.2 | 3,039 | 22.9 | 2.59 | 15.2 |
| 60-69 | 7,963 | 17.4 | 5,758 | 17.7 | 2,205 | 16.6 | 2.61 | 11.7 |
| 70-79 | 4,850 | 10.6 | 3,503 | 10.8 | 1,347 | 10.1 | 2.60 | 9.7 |
| 80-89 | 1,548 | 3.4 | 1,041 | 3.2 | 507 | 3.8 | 2.05 | 5.7 |
| ≥90 | 110 | 0.2 | 63 | 0.2 | 47 | 0.4 | 1.34 |  |
| **Total** | 45,783 | 100 | 32,512 | 100 | 13,271 | 100 | 2.45 | 100 |

**Supplementary Table 1** Stones submitted for analysis by age and sex (n = 45,783)

^a^ The values represent the mean percentage of the German population between 2007 and 2020 for each age group determined by the German census (age 80 and above combined) [14]

Abbreviation: M/F; male-to-female ratio
